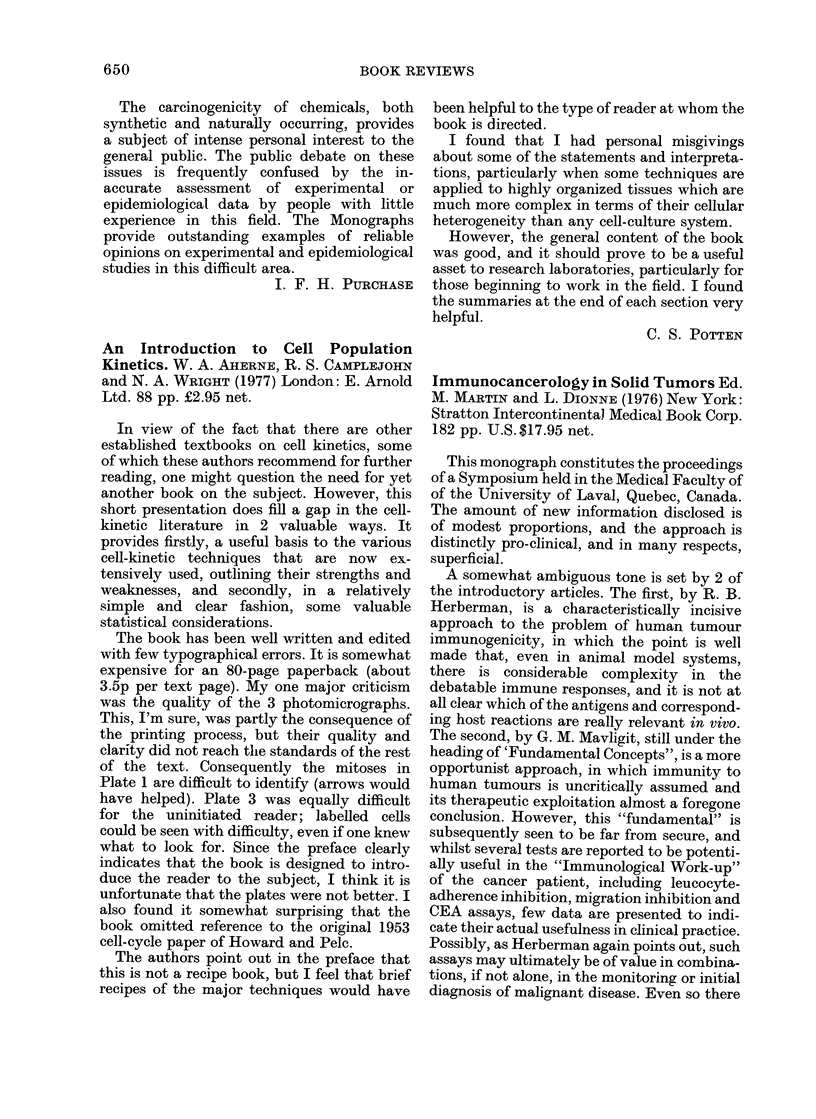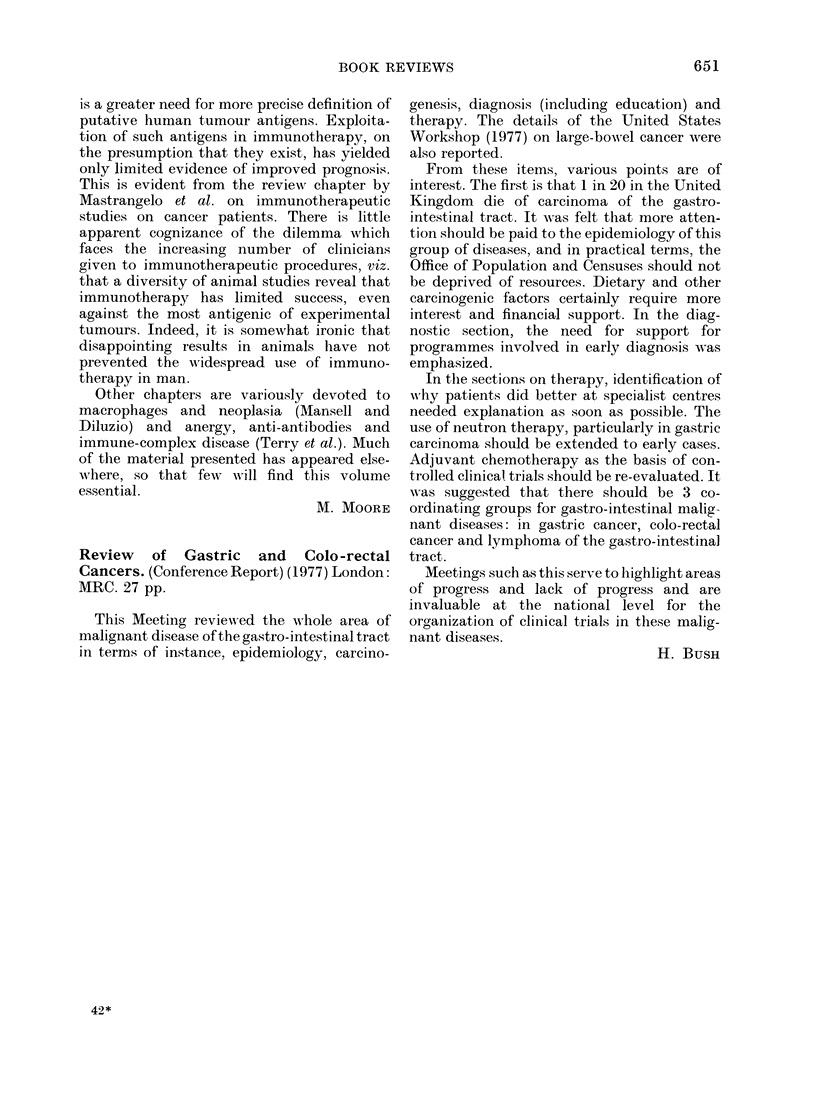# Immunocancerology in Solid Tumours

**Published:** 1978-04

**Authors:** M. Moore


**Immunocancerology in Solid Tumors** Ed. M. MARTIN and L. DIONNE (1976) New York: Stratton Intercontinental Medical Book Corp. 182 pp. U.S.$17.95 net.

This monograph constitutes the proceedings of a Symposium held in the Medical Faculty of of the University of Laval, Quebec, Canada. The amount of new information disclosed is of modest proportions, and the approach is distinctly pro-clinical, and in many respects, superficial.

A somewhat ambiguous tone is set by 2 of the introductory articles. The first, by R. B. Herberman, is a characteristically incisive approach to the problem of human tumour immunogenicity, in which the point is well made that, even in animal model systems, there is considerable complexity in the debatable immune responses, and it is not at all clear which of the antigens and corresponding host reactions are really relevant *in vivo*. The second, by G. M. Mavligit, still under the heading of 'Fundamental Concepts'', is a more opportunist approach, in which immunity to human tumours is uncritically assumed and its therapeutic exploitation almost a foregone conclusion. However, this "fundamental" is subsequently seen to be far from secure, and whilst several tests are reported to be potentially useful in the "Immunological Work-up" of the cancer patient, including leucocyte-adherence inhibition, migration inhibition and CEA assays, few data are presented to indicate their actual usefulness in clinical practice. Possibly, as Herberman again points out, such assays may ultimately be of value in combinations, if not alone, in the monitoring or initial diagnosis of malignant disease. Even so there is a greater need for more precise definition of putative human tumour antigens. Exploitation of such antigens in immunotherapy, on the presumption that they exist, has yielded only limited evidence of improved prognosis. This is evident from the review chapter by Mastrangelo *et al.* on immunotherapeutic studies on cancer patients. There is little apparent cognizance of the dilemma which faces the increasing number of clinicians given to immunotherapeutic procedures, *viz.* that a diversity of animal studies reveal that immunotherapy has limited success, even against the most antigenic of experimental tumours. Indeed, it is somewhat ironic that disappointing results in animals have not prevented the widespread use of immunotherapy in man.

Other chapters are variously devoted to macrophages and neoplasia (Mansell and Diluzio) and anergy, anti-antibodies and immune-complex disease (Terry *et al.*). Much of the material presented has appeared elsewhere, so that few will find this volume essential.

M. MOORE